# Selenoprotein P and type 2 diabetes risk in a large cohort of older German adults

**DOI:** 10.1016/j.redox.2026.104463

**Published:** 2026-10-29

**Authors:** Zhixin Cui, Lutz Schomburg, Lei Peng, Bernd Holleczek, Sandra Hybsier, Josef Köhrle, Hermann Brenner, Ben Schöttker

**Affiliations:** a Division of Clinical Epidemiology of Early Cancer Detection, German Cancer Research Center (DKFZ), Im Neuenheimer Feld 581, 69120 Heidelberg, Germany; b Medical Faculty Heidelberg, Heidelberg University, Im Neuenheimer Feld 672, 69120 Heidelberg, Germany; c Institute of Experimental Endocrinology, Max Rubner Center (MRC) for Cardiovascular Metabolic Renal Research, Charité University Medicine Berlin, CCM, Hessische Straße 4A, 10115 Berlin, Germany; d Saarland Cancer Registry, Neugeländstraße 9, 66117 Saarbrücken, Germany; e Cancer Prevention Graduate School (DKFZ), Im Neuenheimer Feld 280, 69120 Heidelberg, Germany; f Network Aging Research, Heidelberg University, Bergheimer Strasse 20, 69115 Heidelberg, Germany

**Keywords:** Selenoprotein P, Selenium, Diabetes mellitus, Type 2, Cohort study

## Abstract

**Background:**

A previous meta-analysis reported an increased type 2 diabetes (T2D) risk at high blood selenium levels but included few prospective cohorts. Here, we assessed the long-term associations of serum SELENOP concentrations and incident T2D in a large cohort.

**Methods:**

Serum SELENOP concentrations were measured at baseline and 5-year follow-up in 6529 participants aged 50-74 years in the German ESTHER study. Multivariable Cox models were performed to estimate hazard ratios (HRs) and 95% confidence intervals (CIs) for incident T2D across quintiles (Q) of SELENOP concentrations.

**Results:**

During a median follow-up of 11.5 years (interquartile range, 7.1–16.7 years), 1235 participants developed T2D. No statistically significant association between time-dependently modelled SELENOP and T2D risk was observed (HR [95%CI] for Q5 vs. Q1, 1.08 [0.89-1.30]). SELENOP concentrations decreased on average by 0.90 mg/L from baseline to 5-year follow-up. For those 80% of the population, for whom SELENOP decreased by less than 1.83 mg/L, a statistically significant 22% lower T2D risk was observed compared to those 20% with a larger SELENOP decrease (HR [95%CI]: 0.78 [0.62-0.98]). Results were consistent across subgroups defined by sex, age, BMI, smoking status, physical activity level, prediabetes, oxidative stress, inflammatory status, and vitamin D status.

**Conclusions:**

This large prospective cohort study found no increased long-term risk of T2D among older adults residing in Germany with time-dependently modelled SELENOP concentrations. However, although a decrease in SELENOP with ageing is normal, the 20% of the population with the greatest decline in serum SELENOP concentrations showed an increased T2D risk.

## Graphical abstract

Created in BioRender. Schöttker, B. (2026). https://BioRender.com/uhk9brn

**Figure:**
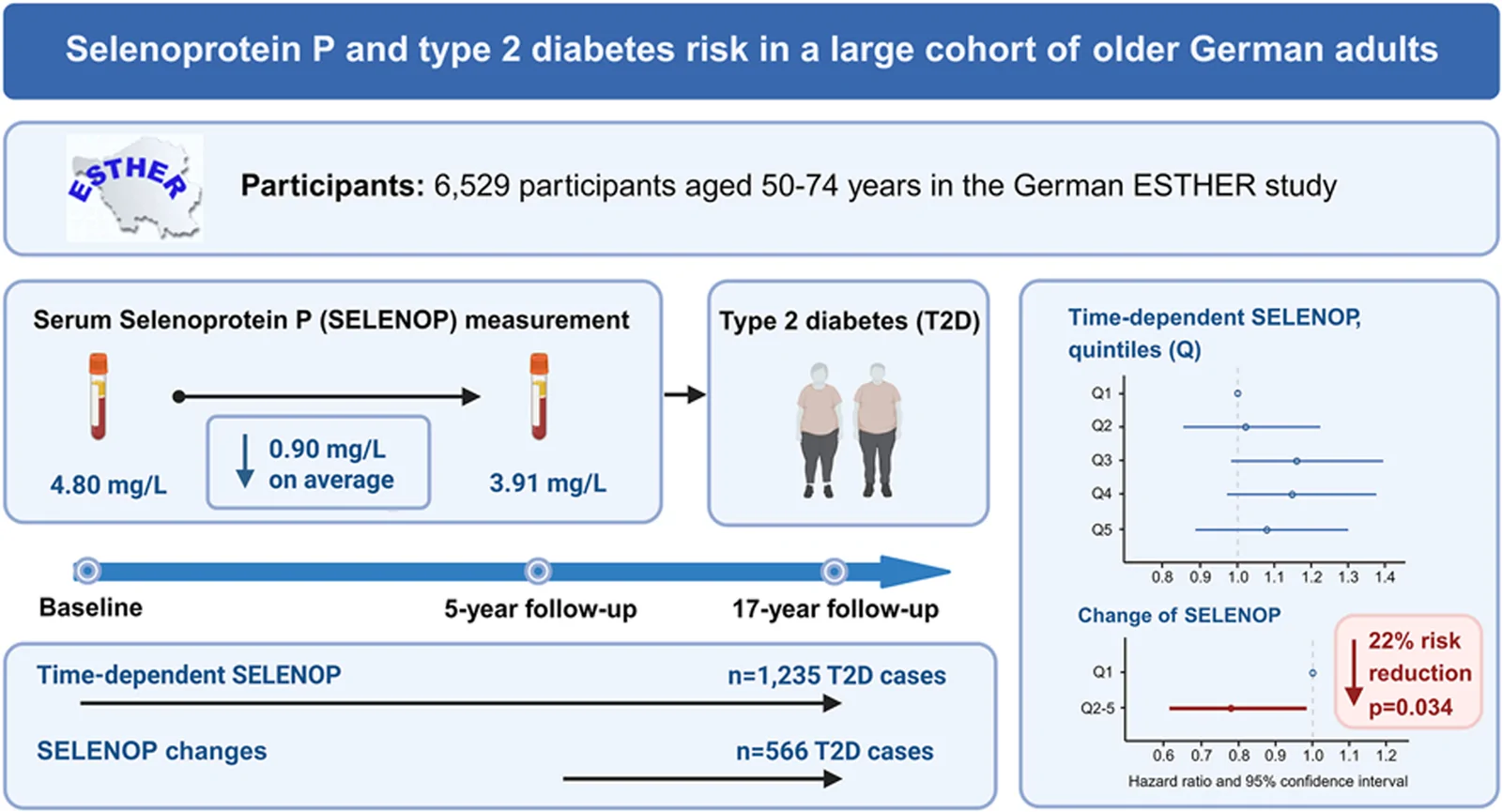

## 1 Introduction

Selenium (Se) is an essential trace element that has important physiological functions through its incorporation into selenoproteins [1]. Several biomarkers have been used to assess Se status, including Se concentrations in serum/plasma, whole blood, urine, toenails, glutathione peroxidase (GPX) activity and selenoprotein P (SELENOP) concentrations [2]. Of note, circulating SELENOP is an important selenoprotein that accounts for more than 50% of circulating Se, reflecting the proportion of retained Se in functional forms, and is considered a sensitive biomarker of Se status, particularly at suboptimal Se status [3]. It is mainly generated in the liver and acts as a Se transporter, delivering Se to target tissues for selenoprotein synthesis, such as the antioxidant GPXs [3], which detoxify peroxides and therefore protect cells from oxidative damage [4]. As oxidative stress plays an important role in the pathogenesis of type 2 diabetes (T2D), which is characterized by chronic hyperglycemia due to insulin resistance and β-cell dysfunction [5], Se status may be associated with the development of T2D.

However, the relationship of Se status with glucose metabolism and insulin sensitivity seemed to be biologically complex. On the one hand, GPX1–3 play important roles in detoxifying hydrogen peroxide and maintaining redox homeostasis in cells [6], including pancreatic β-cells, which are particularly vulnerable to oxidative damage [7], [8]. On the other hand, animal studies also showed that transgenic mice overexpressing GPx1 developed insulin resistance and hyperinsulinemia [9], [10], whereas the knock-out of GPX1 improved insulin-induced glucose uptake and insulin resistance [11]. Furthermore, treatment of mice with purified SELENOP was also shown to impair insulin signaling in the liver and skeletal muscle and to disrupt glucose metabolism [12].

A review and meta-analysis published in 2025 on the association between body Se status and diabetes pooled 72 observational studies and reported that higher urine Se and blood Se were associated with an increased risk of T2D, and a null association was observed for dietary Se intake and nail Se [13]. However, all 4 studies on urine Se were cross-sectional, and only 5 prospective cohort studies with blood Se measurement were included. Among the 5 cohort studies, only 2 had relatively large sample size: the EPIC-Potsdam cohort (n = 2741, participants from the general population) and the China Stroke Primary Prevention Trial (CSPPT) (n = 2,367, hypertension patients) [14], [15]. They both reported a positive association between blood Se and T2D in follow-up periods of no more than seven years. More recently, a study published in 2026 also reported a positive association between serum Se and incident T2D in 1547 participants in a follow-up of up to 4 years [16]. Further studies with larger sample sizes and longer follow-up times are needed because effect size differences by sex and other potential effect-modifying factors remain insufficiently investigated. Moreover, no study has yet examined whether changes in SELENOP or Se concentrations over time are associated with T2D incidence.

Therefore, we aimed to clarify the direction of the association of serum SELENOP concentration measurements with T2D in the largest cohort study to date (n = 6529) with long-term follow-up (17 years) in a population with relatively low Se status (Germany). Furthermore, we aimed to assess differences by sex and other potential effect-modifying factors, and to explore whether changes in SELENOP concentrations over time are associated with T2D incidence.

## 2 Methods

### 2.1 Study design and population

This study is based on data from the ESTHER study (“Epidemiologische Studie zu Chancen der Verhütung, Früherkennung und optimierten Therapie chronischer Erkrankungen in der älteren Bevölkerung”). It is an ongoing, population-based cohort study conducted in older adults from Saarland, Germany. Details are provided elsewhere [17], [18]. In brief, 9940 adults aged 50-74 years were recruited by general practitioners (GPs) during routine health examinations between July 2000 and July 2002. Information from questionnaires was completed by participants and their GPs, and blood samples were collected. Questionnaire-based follow-ups were conducted at 2, 5, 8, 11, 14 and 17 years after recruitment. Blood samples were collected in all rounds with the exception of the 2-year follow-up. Mortality data were ascertained through linkage with population registries. The ESTHER study was approved by the ethics committees of the Medical Faculty of the University of Heidelberg and the Medical Association of Saarland. The study is conducted in compliance with the guidelines of the Declaration of Helsinki. Written informed consent was obtained from all study participants.

Of all the 9940 participants recruited at baseline, the following were excluded (Fig. 1): those with missing baseline T2D diagnoses (n = 244); with prevalent T2D (n = 1801); lost to follow-up for T2D (n = 370), who did not provide blood sample at baseline or the 5-year follow-up (n = 192); for whom SELENOP could not be measured in either round (n = 6); and who provided a baseline blood sample between 07/2000 and 01/2001 but none at the 5-year follow-up (n = 798). Participants recruited before February 2001 were excluded from the analysis because a correlation was observed between Se measurements and total protein concentrations in the samples (r = −0.38, p < 0.001), which might have resulted from the storage conditions. No such correlation was observed in serum samples collected thereafter (r = 0.01, p = 0.555) [18]. A total of 6529 participants were included in the final analysis of the association between time-dependent SELENOP and T2D incidence, comprising 2049 participants with baseline SELENOP measurements only, 930 with SELENOP measured only at 5-year follow-up, and 3450 with SELENOP measurements both at baseline and 5-year follow-up.

**Fig. 1:**
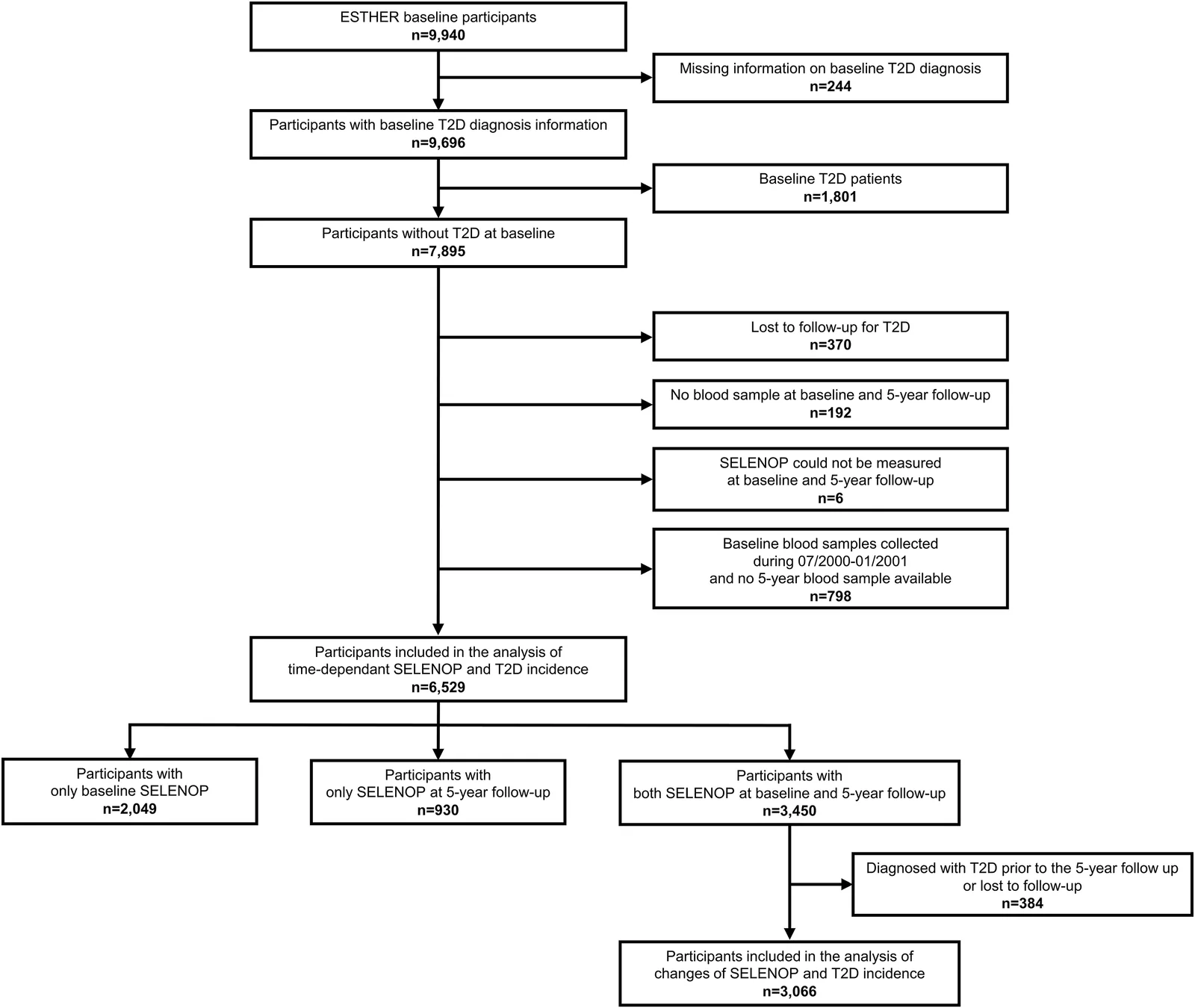
Flow chart of study participants.

Changes in SELENOP were calculated among 3450 participants with SELENOP measurements available at both baseline and the 5-year follow-up. After excluding 384 participants who either developed T2D during the first 5 years of follow-up or were lost to follow-up for T2D after the 5-year follow-up, 3066 participants were included in the analysis of the association between changes in SELENOP concentrations from baseline to the 5-year follow-up and T2D incidence up to the 17-year follow-up.

### 2.2 SELENOP measurement

All serum samples collected were stored at −80°C, and the samples for Se measurement at baseline and 5-year follow-up were transported to the laboratory at the Institute for Experimental Endocrinology, Charité University Medicine, Berlin. Serum SELENOP concentrations were measured using a sandwich enzyme-linked immunosorbent assay (ELISA) method between 2010 and 2012 [19]. Calibration of the ELISA was performed using standard reference material (SRM) 1950 as the reference standard. The SELENOP measurement had a range of 4.8% to 9.3% for the inter-assay coefficient of variation (COV). The intra-assay COV was 8.0%, as determined using diluted human serum [18]. A sample of 59 study participants was randomly selected for additional serum Se measurements, and a strong correlation was observed between serum SELENOP and Se concentrations (r = 0.688, 95% confidence interval (95% CI): 0.518-0.805) [18].

### 2.3 Covariate assessment

Data from the baseline and the 5-year follow-up assessment were used for the analysis. Information on socio-demographic characteristics (age, sex and education), lifestyle and dietary behaviors (smoking status, smoking pack-years, physical activity, regular multivitamin supplement use, dietary intake of fruit, and dietary intake of vegetables), and family history of diabetes was collected from the study participants at both baseline and 5-year follow-up using a comprehensive self-reported questionnaire. Height, weight, and systolic blood pressure were measured by GPs at baseline during the health check-up; weight at the 5-year follow-up was self-reported. Information on lipid-lowering medication and antihypertensive medication at baseline was obtained from medical records from GPs and at the 5-year follow-up from self-reported questionnaires.

Serum triglyceride concentrations (TG) and high-density lipoprotein cholesterol (HDL-C) were measured by enzymatic chromatography; C-reactive protein (CRP) by immunoturbidimetry; and albumin by a fluorescence immunoassay using standard high-throughput procedures of external laboratories. The oxidative stress biomarker, urinary 8-isoprostane, was measured in-house by the 8iso1 enzyme-linked immunosorbent assay (ELISA) kit from Detroit R&D (Detroit, Michigan, USA) as described elsewhere [20]. Serum 25-hydoxyvitamin D (25(OH)D) concentrations were measured by the Diasorin-Liaison immunoassay (Diasorin Inc., Stillwater, USA) among women, and by the IDS-iSYS immunoassay (Immunodiagnostic Systems GmbH, Frankfurt/Main, Germany) among men. Both immunoassay results were standardized to the gold-standard method of liquid chromatography tandem-mass spectrometry, as described in detail elsewhere [21]. Hemoglobin A_1c_ (HbA_1c_) was measured by HPLC with the Bio-Rad Variant II (Bio-Rad, Munich, Germany) at baseline and at the 8-, 11-, and 14-year follow-up.

### 2.4 Type 2 diabetes ascertainment

Type 2 diabetes incidence was ascertained by 1) self- or physician-reported diabetes diagnoses; 2) prescriptions of anti-diabetic drugs; 3) information on fatal diabetes (defined by International Classification of Diseases-10, codes E10-14) from death certificates; or 4) potentially undiagnosed T2D cases defined by HbA_1c_ ≥ 6.5% (48 mmol/mol) as measured at the 8-, 11-, and 14-year follow-up. Sources 1) and 2) were ascertained at the 2-, 5-, 8-, 11-, 14-, and 17-year follow-ups of the ESTHER study, with high response rates among survivors of 96%, 88%, 79%, 67%, 69%, and 58%, respectively. The 17-year follow-up was conducted between mid of 2017 and mid of 2019. Data on the vital status of 99.7% of cohort participants was acquired from the residents' registration offices until end of 2018. The exact dates of the diagnoses were retrieved from their source. If multiple sources indicated a T2D diagnosis, we followed this hierarchy: physicians' questionnaire, participants’ questionnaire, HbA_1c_ measurement date, and date of death. Overall, 3.7% of T2D cases were based only on self-reported information and 7.5% were based only on HbA1c measurements. If no date was provided by either the physician or the study participant, the questionnaire date was used. Overall, the exact date of the T2D diagnosis was unknown for 11.2% of all T2D cases.

### 2.5 Statistical analyses

Baseline characteristics of the study population were summarized as mean ± standard deviation (SD) for normally distributed continuous variables, median and interquartile range (IQR) for non-normally distributed continuous variables, and number and percentage for categorical variables.

The proportional hazards assumption was checked with Schoenfelder residuals, and it was not violated. Therefore, multivariable Cox proportional hazards models were employed to assess the association between serum SELENOP concentrations and T2D incidence. Both baseline and 5-year follow-up serum SELENOP measurements were categorized as quintiles separately, using the lowest quintiles as the reference. Three models were conducted. Model 1 adjusted for age and sex, model 2 additionally adjusted for education, family history of diabetes, systolic blood pressure, body mass index (BMI), smoking status, smoking pack-years, physical activity, regular multivitamin supplement use, dietary intake of fruit, dietary intake of vegetables, lipid-lowering medication, antihypertensive medication, HbA_1c_, TG to HDL-C ratio, and albumin, and model 3 further adjusted for CRP, 8-isoprostane, 25(OH)D concentrations, and season of blood collection. For those with both baseline and 5-year follow-up data, the SELENOP quintiles and covariates were used as time-dependent variables in the models (using either the baseline or 5-year follow-up values depending on the time of the T2D diagnosis prior or after the 5-year follow-up). If the 5-year follow-up measurement was missing, SELENOP was not modelled time-dependently. If the baseline measurement was missing, follow-up started from the 5-year follow-up SELENOP measurement onward. Covariates only assessed at baseline were not modelled time-dependently. These were sex, education, family history of diabetes, smoking pack-years, systolic blood pressure, HbA_1c_, and 8-isoprostane.

A sensitivity analysis was conducted by restricting the follow-up period to the first 5 years to assess the potential influence of reverse causation on the association between baseline serum SELENOP concentrations and incident T2D. The dose-response association between baseline SELENOP concentrations and T2D incidence was assessed using restricted cubic splines with five knots at the 10th, 25th, 50th, 75th, and 90th percentiles of baseline SELENOP concentrations.

The same methods as for the main analysis were used to assess the association between changes in serum SELENOP concentrations from baseline to 5-year follow-up and T2D incidence. However, covariates assessed at the 5-year follow-up were used where available (see above), model 3 was additionally adjusted for baseline SELENOP concentrations, and the T2D follow-up time started at the SELENOP measurement date at the 5-year follow-up. A similar sensitivity analysis by restricting the follow-up period to the first 5 years was also performed.

Subgroup analyses were conducted to assess the association of serum SELENOP concentrations with incident T2D stratified by sex, age group (<65 or ≥65 years), BMI (<25, 25-<30 or ≥30 kg/m^2^), smoking status (never/former-smoker or current smoker), physical activity (“inactive” or “low”, < 2 h each of vigorous and light physical activity per week; or “medium or high”, ≥ 2 h per week of both), prediabetes status (No, HbA_1c_ <5.7%; Yes, HbA_1c_ 5.7-6.4%) [22], inflammatory status (No, CRP <3 mg/L; Yes, CRP ≥ 3 mg/L) [23], urinary 8-isoprostane levels (< median or ≥ median), and 25(OH)D levels (deficient, 25(OH)D < 30 nmol/L; insufficient, 25(OH)D ≥ 30 to <50 nmol/L; or sufficient, 25(OH)D ≥ 50 nmol/L). Furthermore, tests for interactions were conducted using product terms of these baseline characteristics and the continuous SELENOP variable.

Multiple imputation was performed using the fully conditional specification method implemented in the SAS PROC MI procedure to impute missing covariate values at baseline and 5-year follow-up, under the assumption of missing at random. The variables shown in Table 1, except for SELENOP concentrations, were included in the imputation model. The proportion of imputed missing values ranged from 0 to 8.1% (Table 1). Five imputed datasets were generated, and regression analyses were performed on each dataset, with results pooled using the SAS PROC MIANALYZE procedure.

**Table 1:** Description of the baseline characteristics of the study population.

Baseline characteristics	n_total_	n (%)	Mean ± SD or Median [IQR]^a^
Age (mean ± SD**)**	6529		61.8 ± 6.6
Sex (Male)	6529	2791 (42.8)	
School education (Years)	6396		
≤9		4665 (73.0)	
10-11		981 (15.3)	
≥12		750 (11.7)	
BMI (kg/m^2^)	6524		
<25		1980 (30.4)	
25 - <30		3139 (48.1)	
≥30		1405 (21.5)	
Smoking behaviour	6367		
Never smoker		3353 (52.7)	
Former smoker		2035 (32.0)	
Current smoker		979 (15.4)	
Smoking pack-years (median [IQR])	5999		0 [0-18]
Physical activity^b^	6517		
Inactive		1259 (19.3)	
Low		2977 (45.7)	
Medium or high		2281 (35.0)	
Multivitamin supplements	6398	980 (15.3)	
Family history of diabetes	6427	2289 (35.6)	
Lipid-lowering medication	6516	680 (10.4)	
Antihypertensive medication	6516	2573 (39.5)	
Nutrition (median [IQR] portions per day)			
Fruit	6345		1.0 [0.5-3.0]
Vegetables	6237		1.0 [1.0-1.5]
Systolic blood pressure (mmHg), (median [IQR])	6386		140.0 [125.0-150.0]
Triglycerides (mg/dl), (median [IQR])	6518		111.4 [78.3-160.5]
HDL-C (mg/dl), (median [IQR])	6278		53.8 [45.0-63.9]
Albumin (g/L), (mean ± SD**)**	6506		48.7 ± 6.4
25(OH)D concentrations (nmol/L), (median [IQR])	6364		46.3 [35.1-62.4]
C-reactive protein (mg/L), (median [IQR])	6428		2.0 [0.9-4.2]
8-isoprostane (nmol/mmol creatinine), (median [IQR])	6416		0.202 [0.155-0.270]
HbA_1c_ (%), (median [IQR])	6466		5.5 [5.3-5.8]
SELENOP (mg/L), (mean ± SD)	5499		4.80 ± 1.33

Abbreviations: 25-hydoxyvitamin D, 25(OH)D; BMI, body mass index; HDL-C, high density lipoprotein cholesterol; IQR, interquartile range; n_total_, number of participants; SELENOP, selenoprotein P; SD, standard deviation.

a The mean ± SD was used for normally distributed variables (age, albumin and SELENOP) and the median [IQR] for not normally distributed variables (smoking pack-years, portions per day of fruit and vegetables, systolic blood pressure, triglycerides, HDL-C, 25(OH)D, C-reactive protein, 8-isoprostane and HbA_1c_).

b Physical activity levels: “Inactive”, < 1 h each of vigorous and light physical activity per week; “Medium or High”, ≥ 2 h each of vigorous and light physical activity per week. All other levels of physical activity were categorized as “Low”.

All analyses were conducted using SAS, version 9.4 (Cary, North Carolina, USA). A two-tailed p-value < 0.05 was considered statistically significant in the main analyses in the total study population. Among subgroup analyses, the false discovery rate (FDR) < 0.05 was used to indicate statistical significance to account for the increased potential of false-positive findings due to multiple comparisons.

## 3 Results

### 3.1 Baseline characteristics of the study population

Baseline characteristics of the included 6529 participants are shown in Table 1. At baseline, the mean age was 62 years (standard deviation (SD), 6.6 years), and 43% were male. No substantial differences in baseline characteristics of participants included (n = 3066) and excluded (n = 3463) from the analysis on the change of SELENOP levels were detected (Appendix Table 1).

### 3.2 SELENOP distribution

The baseline serum SELENOP concentration was quite normally distributed in 5499 participants, and the mean was 4.80 (SD, 1.33) mg/L (Appendix Fig. 1). At the 5-year follow-up, serum SELENOP was measured in 4380 participants, with a mean concentration of 3.91 (SD, 0.92) mg/L. Among the 3066 participants with both baseline and 5-year follow-up SELENOP measurements, the mean change in serum SELENOP concentration from baseline to the 5-year follow-up was −0.90 (SD, 1.36) mg/L, and the values were approximately normally distributed as well (Appendix Fig. 2). For 22.2% of the study participants, SELENOP concentrations increased.

### 3.3 Association between the SELENOP concentration and T2D incidence

Over 17 years of follow-up (median [interquartile range (IQR)]): 11.5 [7.1-16.7]), 1235 (18.9%) participants developed T2D. In time-dependent Cox regression analyses, no statistically significant association between SELENOP concentrations and T2D risk was observed in any of the models 1 to 3. In the fully adjusted model 3, the hazard ratio (HR) comparing the highest with the lowest SELENOP quintile was 1.08 (95% CI, 0.89-1.30; Table 2). A sensitivity analysis was conducted by restricting the follow-up period to the first 5 years. Similarly, no association was observed between baseline SELENOP and incident T2D (HR [95% CI] in model 3: 1.10 [0.72-1.66]; results were not shown in the tables). These findings were also consistent with the dose-response relationship observed in the restricted cubic spline analysis, which showed no statistically significant association between baseline SELENOP concentrations and T2D risk (Fig. 2).

**Table 2:** Association of serum selenoprotein P concentrations with type 2 diabetes incidence.

SELENOP categories^a^	N_total_	N_cases_	Model 1^b^		Model 2^c^		Model 3^d^	
			HR (95%CI)	*P*	HR (95%CI)	*P*	HR (95%CI)	*P*
Q1	1317	218	Ref		Ref		Ref	
Q2	1301	229	1.00 (0.83; 1.20)	0.984	1.01 (0.84; 1.22)	0.899	1.02 (0.85; 1.23)	0.825
Q3	1319	273	1.18 (0.99; 1.41)	0.072	1.16 (0.97; 1.38)	0.114	1.17 (0.98; 1.40)	0.087
Q4	1293	256	1.10 (0.92; 1.32)	0.291	1.12 (0.93; 1.34)	0.228	1.15 (0.96; 1.38)	0.140
Q5	1299	259	1.13 (0.95; 1.36)	0.174	1.05 (0.87; 1.26)	0.623	1.08 (0.89; 1.30)	0.431

Abbreviations: 25-hydoxyvitamin D, 25(OH)D; BMI, body mass index; CI, confidence interval; CRP, C-reactive protein; HDL-C, high density lipoprotein cholesterol; HbA_1c_: hemoglobin A_1c_; HR, hazard ratio; Q, quintiles; SELENOP, selenoprotein P; TG, triglyceride.

a Cut-offs for the quiniles of serum SELENOP concentrations at baseline: Q1: < 3.74 mg/L; Q2: 3.74 - <4.38 mg/L; Q3: 4.38 - <4.99 mg/L; Q4: 4.99 - <5.74 mg/L; Q5: ≥5.74 mg/L. Cut-offs for the quintiles of serum SELENOP concentrations at 5-year follow-up: Q1: < 3.18 mg/L; Q2: 3.18 - <3.66 mg/L; Q3: 3.66 - <4.10 mg/L; Q4: 4.10 - <4.59 mg/L; Q5: ≥4.59 mg/L.

b Adjusted for age and sex.

c Adjusted for model 1 + education, family history of diabetes, systolic blood pressure, BMI, smoking status, smoking pack-years, physical activity, regular multivitamin supplement use, dietary intake of fruit, dietary intake of vegetables, lipid-lowering medication, antihypertensive medication, HbA_1c_, TG to HDL-C ratio, and albumin.

d Adjusted for model 2 + CRP, 8-isoprostane, 25(OH)D concentrations and season of blood draw.

**Fig. 2:**
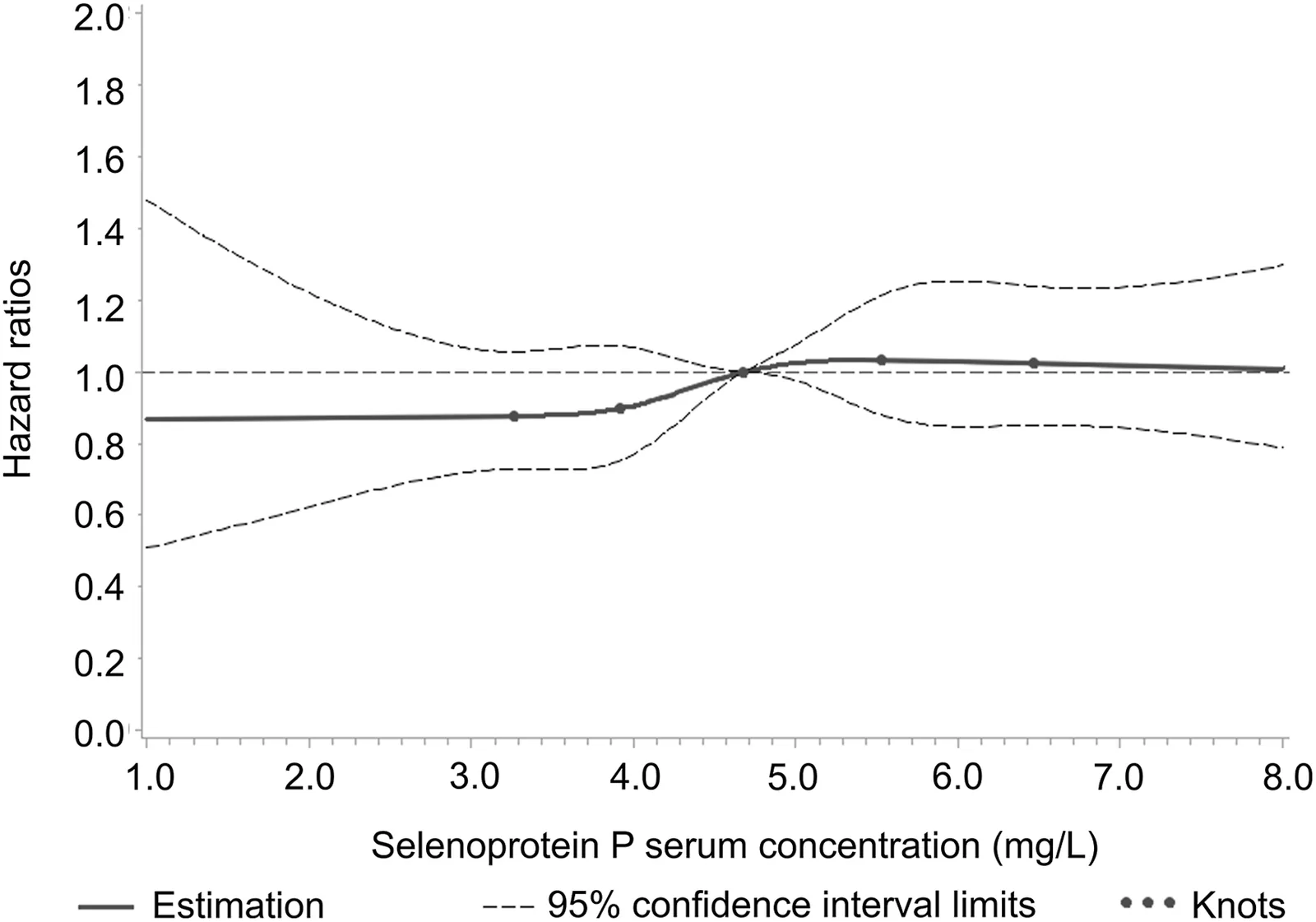
Association of baseline selenoprotein P concentrations with incidence of T2D. Abbreviations: 25-hydoxyvitamin D, 25(OH)D; BMI, body mass index; CRP, C-reactive protein; HDL-C, high density lipoprotein cholesterol; HbA_1c_: hemoglobin A_1c_; SELENOP, selenoprotein P; TG, triglyceride. Note: Restricted cubic spline regression was used to assess the dose-response association between baseline SELENOP concentrations and incident T2D. The model was adjusted for age, sex, education, family history of diabetes, systolic blood pressure, BMI, smoking status, smoking pack-years, physical activity, multivitamin supplement use, dietary intake of fruit and vegetables, lipid-lowering medication, antihypertensive medication, HbA_1c_, TG to HDL-C ratio, albumin, CRP, 8-isoprostane, 25(OH)D concentrations, and season of blood collection. The reference value was set at the median baseline SELENOP concentration and the knots at the 10th, 25th, 50th, 75th, and 90th percentiles of baseline SELENOP concentrations.

In subgroup analyses, no statistically significant association was observed in either men or women after correction for multiple testing using the FDR (Table 3). In addition, similar null results were observed across subgroups defined by age, BMI, smoking status, physical activity, prediabetes status, inflammatory status, oxidative stress (urinary 8-isoprostane levels), and vitamin D status (serum 25(OH)D levels). None of the interaction tests was statistically significant (Appendix Table 2).

**Table 3:** Subgroup analysis for the association of serum selenoprotein P with type 2 diabetes incidence.

Subgroups	Q1^a^			Q2				Q3				Q4				Q5			
	N_total_	N_cases_	HR (95%CI)^b^	N_total_	N_cases_	HR (95%CI)	FDR	N_total_	N_cases_	HR (95%CI)	FDR	N_total_	N_cases_	HR (95%CI)	FDR	N_total_	N_cases_	HR (95%CI)	FDR
**Sex**																			
Male	675	111	1 (ref)	569	108	1.08 (0.82; 1.42)	1.000	534	115	1.23 (0.94; 1.60)	0.345	505	116	1.37 (1.05; 1.80)	0.300	508	104	1.08 (0.82; 1.43)	0.840
Female	642	107	1 (ref)	732	121	1.04 (0.80; 1.35)	1.000	785	158	1.19 (0.93; 1.53)	0.365	788	140	1.08 (0.83; 1.39)	0.760	791	155	1.16 (0.90; 1.49)	0.628
**Age, years**																			
<65	765	120	1 (ref)	748	130	0.99 (0.74; 1.32)	1.000	695	146	1.13 (0.86; 1.48)	0.452	670	138	1.04 (0.79; 1.37)	0.928	675	130	1.09 (0.83; 1.43)	0.830
≥65	552	98	1 (ref)	553	99	1.09 (0.84; 1.40)	1.000	624	127	1.25 (0.98; 1.60)	0.311	623	118	1.32 (1.03; 1.70)	0.308	624	129	1.16 (0.90; 1.51)	0.629
**BMI, kg/m^2^**																			
<25	444	38	1 (ref)	369	35	1.22 (0.76; 1.95)	1.000	406	40	1.14 (0.72; 1.81)	0.616	364	44	1.33 (0.85; 2.09)	0.423	401	42	1.11 (0.70; 1.76)	0.841
25 - <30	568	86	1 (ref)	666	118	1.19 (0.90; 1.58)	1.000	619	140	1.39 (1.06; 1.82)	0.275	610	115	1.23 (0.93; 1.64)	0.362	587	122	1.32 (1.00; 1.76)	0.415
≥30	305	94	1 (ref)	266	76	0.85 (0.62; 1.16)	1.000	294	93	0.93 (0.69; 1.26)	0.649	319	97	1.00 (0.74; 1.34)	0.997	311	95	0.87 (0.64; 1.17)	0.795
**Smoking status**																			
Never/former smoker	1105	187	1 (ref)	1132	194	0.99 (0.81; 1.22)	1.000	1154	238	1.15 (0.95; 1.40)	0.360	1141	231	1.20 (0.98; 1.46)	0.354	1158	236	1.11 (0.91; 1.36)	0.683
Current smoker	212	31	1 (ref)	169	35	1.45 (0.88; 2.40)	1.000	165	35	1.54 (0.92; 2.57)	0.327	152	25	1.04 (0.60; 1.81)	0.962	141	23	1.45 (0.81; 2.61)	0.614
**Physical activity**^c^																			
Inactive or low	836	147	1 (ref)	777	135	1.01 (0.79; 1.28)	1.000	730	151	1.11 (0.88; 1.40)	0.433	708	142	1.10 (0.87; 1.40)	0.599	713	143	1.05 (0.82; 1.33)	0.867
Medium or High	481	71	1 (ref)	524	94	1.12 (0.82; 1.54)	1.000	589	122	1.38 (1.02; 1.86)	0.287	585	114	1.34 (0.99; 1.83)	0.310	586	116	1.32 (0.97; 1.78)	0.487
**Prediabetes**^d^																			
No	814	87	1 (ref)	827	86	0.91 (0.67; 1.23)	1.000	800	108	1.25 (0.94; 1.67)	0.340	805	95	1.03 (0.77; 1.39)	0.944	776	95	1.06 (0.79; 1.44)	0.856
Yes	503	131	1 (ref)	474	143	1.14 (0.90; 1.46)	1.000	519	165	1.23 (0.97; 1.56)	0.324	488	161	1.25 (0.99; 1.59)	0.344	523	164	1.23 (0.97; 1.56)	0.540
**Inflammation**^e^																			
No	1119	199	1 (ref)	1131	206	1.02 (0.84; 1.24)	1.000	1168	244	1.15 (0.95; 1.40)	0.353	1140	229	1.13 (0.93; 1.37)	0.423	1139	230	1.07 (0.88; 1.30)	0.814
Yes	198	19	1 (ref)	170	23	1.15 (0.62; 2.16)	1.000	151	29	1.56 (0.84; 2.88)	0.363	153	27	1.52 (0.81; 2.85)	0.400	160	29	1.49 (0.80; 2.77)	0.576
**8-isoprostane**																			
< median	585	90	1 (ref)	635	101	1.11 (0.83; 1.48)	1.000	694	141	1.29 (0.99; 1.69)	0.304	657	110	1.21 (0.91; 1.61)	0.380	693	132	1.34 (1.01; 1.76)	0.396
≥ median	732	128	1 (ref)	666	128	0.99 (0.77; 1.28)	0.965	625	132	1.13 (0.88; 1.45)	0.413	636	146	1.19 (0.93; 1.53)	0.380	606	127	0.98 (0.76; 1.26)	0.903
**25 (OH)D levels**^f^																			
Deficient	255	41	1 (ref)	181	31	1.07 (0.65; 1.78)	1.000	167	38	1.31 (0.82; 2.10)	0.369	150	40	1.41 (0.88; 2.24)	0.372	140	30	1.06 (0.65; 1.75)	0.902
Insufficient	497	90	1 (ref)	488	93	0.98 (0.73; 1.32)	1.000	496	108	1.18 (0.88; 1.57)	0.400	489	89	1.04 (0.77; 1.40)	0.942	482	90	1.01 (0.75; 1.38)	0.932
Sufficient	565	87	1 (ref)	632	105	1.07 (0.80; 1.43)	1.000	656	127	1.18 (0.90; 1.56)	0.369	654	127	1.24 (0.94; 1.64)	0.357	677	139	1.26 (0.96; 1.67)	0.562

Abbreviations: 25-hydoxyvitamin D, 25(OH)D; BMI, body mass index; CI, confidence interval; CRP, C-reactive protein; HDL-C, high density lipoprotein cholesterol; HbA_1c_: hemoglobin A_1c_; HR, hazard ratio; Q, quintiles; SELENOP, selenoprotein P; TG, triglyceride.

a Cut-offs for the quintiles of serum SELENOP concentrations at baseline: Q1: < 3.74 mg/L; Q2: 3.74 - <4.38 mg/L; Q3: 4.38 - <4.99 mg/L; Q4: 4.99 - <5.74 mg/L; Q5: ≥5.74 mg/L. Cut-offs for the quintiles of serum SELENOP concentrations at 5-year follow-up: Q1: < 3.18 mg/L; Q2: 3.18 - <3.66 mg/L; Q3: 3.66 - <4.10 mg/L; Q4: 4.10 - <4.59 mg/L; Q5: ≥4.59 mg/L.

b Adjusted for age and sex, education, family history of diabetes, systolic blood pressure, BMI, smoking status, smoking pack-years, physical activity, regular multivitamin supplement use, dietary intake of fruit, dietary intake of vegetables, lipid-lowering medication, antihypertensive medication, HbA_1c_, TG to HDL-C ratio, albumin, CRP, 8-isoprostane, 25(OH)D concentrations and season of blood draw.

c “Inactive” or “low”, < 2 h each of vigorous and light physical activity per week; or “medium or high”, ≥ 2 h per week of both.

d No, HbA_1c_ <5.7%; or Yes, HbA_1c_ 5.7-6.4%.

e No, CRP <3 mg/L; Yes, CRP ≥ 3 mg/L.

f Deficient, 25(OH)D < 30 nmol/L; insufficient, 25(OH)D 30 to <50 nmol/L; or sufficient, 25(OH)D ≥ 50 nmol/L.

In an additional analysis, we used the change in SELENOP concentrations from baseline to 5-year follow-up as the exposure, and the follow-up time started at the 5-year follow-up. This analysis included 3066 study participants, of whom 566 developed T2D during a median follow-up time of 10.2 years (IQR, 5.3-11.9 years). Compared with participants in the quintile with the greatest decrease in SELENOP concentrations (Q1), all other quintiles showed inverse HRs for incident T2D, which did not reach statistical significance except for Q3 in models 2 and 3 (Table 4). Therefore, we conducted an additional analysis combining Q2–Q5 as the exposure while keeping Q1 as the reference group. Participants in the combined Q2–Q5 group had a statistically significant 22% lower risk of incident T2D than those in Q1 in the fully adjusted model 3 (HR [95% CI]: 0.78 [0.62-0.98]). In the sensitivity analysis restricting the follow-up period to the first 5 years after the 5-year follow-up, statistical precision was lower due to the smaller number of cases. However, the direction and strength of the association remained unchanged (HR [95% CI] in Model 3 for Q2–Q5 vs. Q1: 0.76 [0.55–1.06]; results not shown in tables).

**Table 4:** Association between the changes of serum selenoprotein P concentrations from baseline to 5-year follow-up and type 2 diabetes incidence in the subsequent up to 12 years.

Quintiles of the change of SELENOP,	N_total_	N_cases_	Model 1^a^		Model 2^b^		Model 3^c^	
mg/L			HR (95%CI)	*P*	HR (95%CI)	*P*	HR (95%CI)	*P*
Q1, < −1.83	613	126	Ref		Ref		Ref	
Q2, -1.83 - < -1.10	613	106	0.83 (0.64; 1.08)	0.166	0.83 (0.64; 1.07)	0.151	0.79 (0.60; 1.06)	0.112
Q3, -1.10 - < -0.54	614	107	0.82 (0.63; 1.06)	0.120	0.75 (0.58; 0.97)	0.030	0.71 (0.53; 0.97)	0.032
Q4, -0.54 - <0.10	613	119	0.95 (0.74; 1.22)	0.683	0.90 (0.70; 1.16)	0.417	0.85 (0.62; 1.17)	0.329
Q5, ≥ 0.10	613	108	0.84 (0.65; 1.09)	0.185	0.78 (0.60; 1.02)	0.068	0.74 (0.52; 1.06)	0.099
Q1, < −1.83	613	126	Ref		Ref		Ref	
Q2-5, ≥ −1.83	2453	440	0.83 (0.69; 1.01)	0.058	0.82 (0.68; 0.99)	0.035	0.78 (0.62; 0.98)	0.034

Abbreviations: 25-hydoxyvitamin D, 25(OH)D; BMI, body mass index; CI, confidence interval; CRP, C-reactive protein; HDL-C, high density lipoprotein cholesterol; HbA_1c_: hemoglobin A_1c_; HR, hazard ratio; Q, quintiles; SELENOP, selenoprotein P; TG, triglyceride.

a Adjusted for age and sex.

b Adjusted for model 1 + education, family history of diabetes, systolic blood pressure, BMI, smoking status, smoking pack-years, physical activity, regular multivitamin supplement use, dietary intake of fruit, dietary intake of vegetables, lipid-lowering medication, antihypertensive medication, HbA_1c_, TG to HDL-C ratio, and albumin.

c Adjusted for model 2 + CRP, 8-isoprostane, 25(OH)D concentrations, season of blood draw and baseline SELENOP concentrations.

## 4 Discussion

In this large prospective cohort study, no statistically significant long-term association was observed between time-dependently modelled serum SELENOP concentrations and incident T2D in the overall population and subgroup analyses. However, the changes in SELENOP concentrations in the first 5 years of follow-up were associated with T2D risk. Those 20% of study participants with the greatest decrease in SELENOP concentrations had a higher T2D risk than the rest of the population.

### 4.1 Comparison with previous studies on the association of SELENOP with T2D

A recent meta-analysis of 9 studies reported no statistically significant difference in SELENOP levels between T2D cases and controls [24]. However, results were not adjusted for potential confounders. Therefore, we would like to highlight the results of the two largest studies, which assessed adjusted risk estimates for the association between SELENOP and T2D [14], [25]. The cross-sectional Berlin Aging Study II included 1500 participants with a median age of 68 years and a median serum SELENOP concentration of 3.79 mg/L and found no statistically significant association between serum SELENOP concentrations and T2D risk (Odds ratio [95% CI] for per 1 mg/L increase, 1.15 [0.98-1.34]) [25]. However, the Berlin Aging Study II reported a statistically significant inverse association between SELENOP and T2D in men (OR per 1 mg/L increase [95%CI], 1.22 [1.00, 1.48] but not in women (OR [95%CI], 1.08 [0.83-1.41]). However, confidence intervals overlapped, and the test on interaction between SELENOP and sex was not statistically significant (P_interaction_ = 0.5).

A case-cohort study embedded in the EPIC-Potsdam study, including 705 incident T2D cases and 2090 subcohort participants (median age, 49 years; median serum SELENOP concentration, 5.3 mg/L), reported that higher serum SELENOP concentrations were associated with an increased risk of T2D (HR per SD [95% CI], 1.14 [1.02-1.29]) after adjusting for age, sex, education and T2D risk factors over a median follow-up of 6.6 years [14]. The review of M. P. Rayman et al. suggested that the observed association could result from pathophysiological changes associated with diabetes, indicating the possibility of residual confounding or reverse causation [26]. To examine whether the discrepancy between our findings and those of the previous EPIC-Potsdam study could be explained by reverse causation, we restricted the follow-up to the first 5 years. If reverse causation were present, the association between SELENOP and T2D incidence would be expected to be stronger during the early follow-up period. However, this was not the case in our study.

A possible explanation for the discrepant findings between the EPIC Potsdam and ESTHER study is the age difference (median ages of 49 vs. 62 years, respectively), which came along with higher baseline SELENOP concentrations (5.3 vs. 4.8 mg/L, respectively). In our previous publication from the ESTHER study, we showed that age was inversely associated with serum SELENOP levels in both cross-sectional and longitudinal analyses [18]. Maybe, the increased risk of T2D in EPIC-Potsdam among subjects with high SELENOP could not be observed in the older ESTHER participants because the latter cohort included fewer participants with high SELENOP concentrations.

To our knowledge, this is the first study to investigate the association between changes in serum SELENOP concentrations and incident T2D. Participants in the quintile with the greatest decrease had a higher risk of incident T2D, compared with those in other quintiles combined. More large-scale prospective studies are needed to corroborate our findings.

### 4.2 Comparison with previous studies on the association of Se with T2D

Liang XL et al. published a meta-analysis of observational studies on the association of Se and T2D in 2025 [13]. This review included 72 observational studies, with 40 for blood Se, 4 for urine Se, 9 for nail Se, and 20 for dietary Se intake. Higher urine Se and blood Se were associated with an increased risk of T2D, whereas no association was observed for dietary or nail Se. However, all the previous studies on urine Se and most of the studies on blood Se were cross-sectional studies. Previous cohort studies on the association between blood Se and T2D in the general population are limited and inconclusive. Only four cohort studies conducted in the general population were included in the meta-analysis by Liang XL et al. [14], [27], [28], [29], and one additional cohort study was published thereafter [16]. Among these five studies, the EPIC-Potsdam study (n = 705 cases; median Se: 80 μg/L; follow-up time: 6.6 years), and the Chinese cohort of Wang et al. (n = 309 cases; median serum Se: 98.5 μg/L; follow-up time: 3.3 years) reported a positive association between blood Se concentrations and T2D risk in the total population [14], [16]. In contrast, the Swedish Uppsala Longitudinal Study of Adult Men (ULSAM) (n = 88 cases; mean Se: 75.6 μg/L, follow-up time: 20 years), the Spanish Hortega study (n = 75 cases; mean Se: 83.9 μg/L, follow-up time: 13.2 years), and the Chinese nested case–control of Yuan et al. (n = 1515 cases; median Se: 61.7 μg/L among controls and 64.3 μg/L among cases; follow-up time: 4.6 years) observed no association [27], [28], [29]. Whereas the absence of a detected higher T2D risk at higher Se levels in the study of Yuan et al. can be explained by the relatively low baseline Se levels, the null results in the ULSAM and Hortega study may be either explained by the relatively low case numbers or by declined Se blood levels with ageing over the long follow-up periods of these studies [27], [28]).

In terms of previous randomized controlled trials (RCTs), as reviewed by Liang XL et al. [30], six RCTs assessed the effect of Se supplementation on T2D risk. In line with our findings, most of the RCTs did not observe a statistically significant effect of Se supplementation on the occurrence of T2D, such as the Se and vitamin E Cancer Prevention Trial (SELECT) [31], [32] and the Se and Celecoxib Trial (SelCel) [33]. The post-hoc analysis of the Nutritional Prevention of Cancer (NPC) trial reported increased T2D risk with Se supplementation [34]. Of note, NPC trial participants had a history of nonmelanoma skin cancer. Se exposure outside the optimal range may compromise its benefits or even potentially induce adverse effects, particularly in some specific populations residing in areas with high soil Se availability and displaying habitual high Se intakes [16].

### 4.3 Previous mechanistic evidence on Se and T2D

Type 2 diabetes is increasingly recognized as a condition involving chronic low-grade inflammation driven by metabolic dysregulation and oxidative stress [35], [36]. Disrupted redox homeostasis may contribute to insulin resistance and β-cell dysfunction [37], [38]. Many selenoproteins play important roles in antioxidant defense and redox regulation, such as GPXs, SELENOP and thioredoxin reductases (TXNRDs) [1]. However, antioxidants are double-edged swords [39], and both suboptimal Se and overexpression of certain selenoproteins may disrupt glucose metabolism and promote the development of T2D [40]. Some studies showed that overexpression of GPX1 in mice induced hyperglycemia and hyperinsulinemia and was accompanied by reduced insulin-stimulated phosphorylation of the insulin receptor in the liver [9]. Other studies showed that purified SELENOP impaired insulin signaling and disrupted glucose homeostasis in hepatocytes and myocytes, whereas SELENOP gene deletion improved glucose tolerance and insulin sensitivity in mice [12]. In our study of older German adults, the generally low Se status of the participants may explain why the adverse metabolic effects observed with high Se exposure or selenoprotein expression were not observed.

Some selenoproteins were shown to be associated with protective metabolic effects, and their deficiency or impaired function may adversely affect glucose homeostasis. For example, TXNRD inhibition increased the susceptibility of β-cells to hydrogen peroxide-induced cell death [41], and glucose-stimulated insulin secretion was observed to be impaired in mice with a specific knockout of TXNRD1 in their pancreatic β-cells [42]. In addition, disruption of the genes encoding type 2 or type 3 iodothyronine deiodinases (DIO2 or DIO3) in mice was associated with insulin resistance [43] or impaired insulin secretion [44]. Furthermore, from an immunological perspective, selenoprotein F deficiency was shown to be associated with antigen presentation and endoplasmic reticulum-to-Golgi transport, highlighting its potential role in immune-related processes [45]. These mechanisms may explain why we observed in our study that subjects in the quintile with the greatest decrease in SELENOP concentrations in the first 5 years of follow-up had a higher risk of incident T2D than the rest of the study population (quintiles 2-5 combined).

### 4.4 Strengths and limitations

Our study has several strengths. The major strength was that we used repeated measurements of SELENOP concentrations and covariates during the 5-year follow-up. In addition, we carefully adjusted for a wide range of potential confounders and performed comprehensive subgroup analyses.

Several limitations should be considered. The primary limitation is the observational nature of this prospective cohort study. Despite adjustment for a wide range of potential confounders, residual confounding due to unmeasured factors cannot be ruled out. Another limitation is that the absolute SELENOP concentration measurements might be affected by the use of SRM1950 to standardize the SELENOP assay. SRM1950 was developed in a U.S. population with higher serum SELENOP concentrations and a more diverse ethnic composition, whereas the ESTHER study population has lower SELENOP concentrations and is almost exclusively Caucasian. However, this would not affect the estimated HRs and 95% CIs across SELENOP quintiles. Currently, no SRM specifically developed for a European population is available. Furthermore, we could not determine to what extent the observed decline in SELENOP concentrations with aging reflects true biological changes or was a result of different sample storage conditions or batch effects (as we did not measure baseline and 5-year follow-up samples in the same batches). This issue warrants further investigation in future studies. Additionally, as mentioned before, participants in the ESTHER study were older German adults and almost predominantly Caucasian. Therefore, our findings may not be generalizable to other populations.

### 4.5 Conclusion

Our study did not detect an association between time-dependently modelled serum SELENOP concentration and the development of T2D in older German adults. As this is, to our knowledge, the largest prospective cohort study with long-term follow-up to date, our study was able to clarify that neither low nor high SELENOP concentrations are associated with T2D risk. However, the 20% of the study population with the greatest decrease in SELENOP concentrations over 5 years had a statistically significantly increased risk of incident T2D. These findings suggest that the absolute SELENOP concentration is not as relevant as a greater SELENOP decrease over time than can be expected with ageing. If well-designed RCTs can confirm that attenuating the decline in SELENOP concentrations with advancing age by Se supplementation reduces the risk of T2D, regular measurement of serum SELENOP during health check-ups may be warranted to monitor SELENOP changes and treat decreasing levels.

## CRediT authorship contribution statement

**Zhixin Cui:** Writing – review & editing, Writing – original draft, Project administration, Methodology, Investigation, Formal analysis, Conceptualization. **Lutz Schomburg:** Writing – review & editing, Investigation. **Lei Peng:** Writing – review & editing. **Bernd Holleczek:** Writing – review & editing, Resources, Investigation, Data curation. **Sandra Hybsier:** Writing – review & editing, Investigation. **Josef Köhrle:** Writing – review & editing. **Hermann Brenner:** Writing – review & editing, Resources, Investigation, Data curation. **Ben Schöttker:** Writing – review & editing, Supervision, Resources, Investigation, Data curation.

## Funding

The first author is an awardee of a PhD scholarship of the Chinese Scholarship Council. No specific funding was obtained for this project. The SELENOP measurements were carried out in a previous project funded by the German Cancer Aid Foundation (grant reference no: 108250). The ESTHER study was funded by the Baden-Württemberg state Ministry of Science, Research and Arts (Stuttgart, Germany), the 10.13039/501100002347Federal Ministry of Research, Technology and Space (Berlin, Germany), the Federal Ministry of Education, Family Affairs, Senior Citizens, and Youth (Berlin, Germany), and the Saarland Ministry of Labour, Social Affairs, Women and Public Health.

## Declaration of competing interest

L.S. holds shares in selenOmed GmbH, Berlin, a company involved in Se status assessment. All other authors have no conflicts of interest.

## Data Availability

Data will be made available on request.
