# Supplementary material for: Selenoprotein P and type 2 diabetes risk in a large cohort of older German adults
Source: Redox Biol. 2026 Oct 29;98:104463. doi: 10.1016/j.redox.2026.104463 (PMC13763019; doi:10.1016/j.redox.2026.104463)
Supplement: Supplementary file 1 [file mmc1.docx]

**Appendix to the Article:**

**“Selenoprotein P and type 2 diabetes risk in a large cohort of**

**older German adults”**

**Contents**

[Appendix Figure 1. Histogram of baseline serum selenoprotein P concentrations	2](#_Toc243278203)

[Appendix Figure 2. Histogram of changes in serum selenoprotein P concentrations over the	first 5-year follow-up	3](#_Toc243278204)

[Appendix Table 1. Comparison of the baseline characteristics of the study populations included	and excluded from the analysis on changes of SELENOP and T2D incidence	4](#_Toc243278205)

[Appendix Table 2. Subgroup analyses and interaction tests for the association of serum	selenoprotein P with type 2 diabetes incidence	6](#_Toc243278206)

Appendix Figure 1. Histogram of baseline serum selenoprotein P concentrations

Appendix Figure 2. Histogram of changes in serum selenoprotein P concentrations over the first 5-year follow-up

# **Appendix Table 1. Comparison of the baseline characteristics of the study populations included and excluded from the analysis on changes of SELENOP and T2D incidence**

| **Baseline characteristics** |  | **Included** |  |  |  | **Excluded** |  |
| --- | --- | --- | --- | --- | --- | --- | --- |
|  | **n_total_** | **n (%)** | **Mean ± SD or**  **Median [IQR]** ^a^ |  | **n_total_** | **n (%)** | **Mean ± SD or**  **Median [IQR]** ^a^ |
| Age (mean **±** SD**)** | 3,066 |  | 62.0 ± 6.4 |  | 3,463 |  | 61.5 ± 6.6 |
| Sex (Male) | 3,066 | 1,338 (43.6) |  |  | 3,463 | 1,453 (42.0) |  |
| School education (Years) | 3,011 |  |  |  | 3,385 |  |  |
| ≤9 |  | 2,202 (73.1) |  |  |  | 2,463 (72.8) |  |
| 10-11 |  | 443 (14.7) |  |  |  | 538 (15.9) |  |
| ≥12 |  | 366 (12.2) |  |  |  | 384 (11.3) |  |
| BMI (kg/m^2^) | 3,062 |  |  |  | 3,462 |  |  |
| <25 |  | 952 (31.1) |  |  |  | 1,028 (29.7) |  |
| 25 - <30 |  | 1,517 (49.5) |  |  |  | 1,622 (46.9) |  |
| ≥30 |  | 593 (19.4) |  |  |  | 812 (23.5) |  |
| Smoking status | 2,982 |  |  |  | 3,385 |  |  |
| Never smoker |  | 1,606 (53.9) |  |  |  | 1,747 (51.6) |  |
| Former smoker |  | 1,000 (33.5) |  |  |  | 1,035 (30.6) |  |
| Current smoker |  | 376 (12.6) |  |  |  | 603 (17.8) |  |
| Smoking pack-years (median [IQR]) | 2,807 |  | 0 [0-20] |  | 3,192 |  | 0 [0-16] |
| Physical activity ^b^ | 3,062 |  |  |  | 3,455 |  |  |
| Inactive |  | 497 (16.2) |  |  |  | 762 (22.0) |  |
| Low |  | 1,409 (46.0) |  |  |  | 1,568 (45.4) |  |
| Medium or high |  | 1,156 (37.8) |  |  |  | 1,125 (32.6) |  |
| Multivitamin supplements | 3,015 | 478 (15.9) |  |  | 3,383 | 502 (14.8) |  |
| Family history of diabetes | 3,027 | 1,091 (36.0) |  |  | 3,400 | 1,198 (35.2) |  |
| Lipid-lowering medication | 3,060 | 348 (11.4) |  |  | 3,456 | 332 (9.6) |  |
| Antihypertensive medication | 3,060 | 1,192 (39.0) |  |  | 3,456 | 1,381 (40.0) |  |
| Nutrition (median [IQR] portions per day) |  |  |  |  |  |  |  |
| Fruit | 2,992 |  | 1.0 [0.5-3.0] |  | 3,353 |  | 1.0 [0.5-3.0] |
| Vegetables | 2,946 |  | 1.0 [1.0-1.5] |  | 3,291 |  | 1.0 [1.0-1.5] |
| Systolic blood pressure (mmHg), (median [IQR]) | 3,006 |  | 140.0 [125.0-150.0] |  | 3,380 |  | 140.0 [128.0-150.0] |
| Triglycerides (mg/dl), (median [IQR]) | 3,063 |  | 111.5 [79.5-158.9] |  | 3,455 |  | 111.4 [77.4-161.1] |
| HDL-C (mg/dl), (median [IQR]) | 3,044 |  | 54.0 [45.2-64.0] |  | 3,234 |  | 53.2 [44.0-63.5] |
| Albumin (g/L), (mean **±** SD**)** | 3,055 |  | 50.1 ± 3.6 |  | 3,451 |  | 47.6 ± 8.0 |
| 25(OH)D concentrations (nmol/L), (median [IQR]) | 2,993 |  | 47.1 [35.9-63.9] |  | 3,371 |  | 45.5 [34.3-61.0] |
| C-reactive protein (mg/L), (median [IQR]) | 3,022 |  | 1.9 [0.9-4.0] |  | 3,406 |  | 2.0 [0.9-4.3] |
| 8-isoprostane (nmol/mmol creatinine), (median [IQR]) | 3,018 |  | 0.198 [0.153-0.265] |  | 3,398 |  | 0.207 [0.157-0.275] |
| HbA_1c_ (%), (median [IQR]) | 3,035 |  | 5.5 [5.3-5.8] |  | 3,431 |  | 5.5 [5.3-5.8] |
| SELENOP (mg/L), (mean **±** SD**)** | 3,066 |  | 4.83 ± 1.35 |  | 2,433 |  | 4.76 ± 1.30 |

Abbreviations: 25-hydoxyvitamin D, 25(OH)D; BMI, body mass index; HbA_1c_: hemoglobin A_1c_; HDL-C, high density lipoprotein cholesterol; IQR, interquartile range; n_total_, number of participants; SELENOP, selenoprotein P; SD, standard deviation.

^a^ The mean ± SD was used for normally distributed variables (age, albumin and SELENOP) and the median [IQR] for not normally distributed variables (smoking pack-years, portions per day of fruit and vegetables, systolic blood pressure, triglycerides, HDL-C, 25(OH)D, C-reactive protein, 8-isoprostane and HbA_1c_).

^b^ Physical activity levels: “Inactive”, < 1 hour each of vigorous and light physical activity per week; “Medium or High”, ≥ 2 hours each of vigorous and light physical activity per week. All other levels of physical activity were categorized as “Low”.

# **Appendix Table 2. Subgroup analyses and interaction tests for the association of serum selenoprotein P with type 2 diabetes incidence**

| **Subgroups** | **N_total_** | **N_cases_** | **HR (95%CI) ^a^** | **FDR** | **P_interaction_ ^b^** |
| --- | --- | --- | --- | --- | --- |
| **Sex** |  |  |  |  | 0.869 |
| Male | 2,791 | 554 | 1.07 (0.98; 1.16) | 0.192 |  |
| Female | 3,738 | 681 | 1.09 (1.01; 1.18) | 0.104 |  |
| **Age, years** |  |  |  |  | 0.886 |
| <65 | 3,553 | 664 | 1.06 (0.98; 1.15) | 0.225 |  |
| ≥65 | 2,976 | 571 | 1.09 (1.01; 1.19) | 0.113 |  |
| **BMI, kg/m^2^** |  |  |  |  | 0.140 |
| <25 | 1,984 | 199 | 1.06 (0.93; 1.22) | 0.446 |  |
| 25 - <30 | 3,050 | 581 | 1.09 (1.00; 1.18) | 0.125 |  |
| ≥30 | 1,495 | 455 | 1.02 (0.93; 1.12) | 0.672 |  |
| **Smoking status** |  |  |  |  | 0.241 |
| Never/former smoker | 5,690 | 1,086 | 1.07 (1.00; 1.13) | 0.120 |  |
| Current smoker | 839 | 149 | 1.22 (1.02; 1.45) | 0.090 |  |
| **Physical activity ^c^** |  |  |  |  | 0.267 |
| Inactive or low | 3,764 | 718 | 1.06 (0.99; 1.14) | 0.176 |  |
| Medium or High | 2,765 | 517 | 1.12 (1.02; 1.22) | 0.080 |  |
| **Prediabetes ^d^** |  |  |  |  | 0.221 |
| No | 4,022 | 471 | 1.04 (0.95; 1.13) | 0.471 |  |
| Yes | 2,507 | 764 | 1.11 (1.04; 1.20) | 0.040 |  |
| **Inflammation ^e^** |  |  |  |  | 0.212 |
| No | 5,697 | 1,108 | 1.06 (1.00; 1.12) | 0.140 |  |
| Yes | 832 | 127 | 1.16 (0.96; 1.39) | 0.189 |  |
| **8-isoprostane** |  |  |  |  | 0.899 |
| < median | 3,264 | 574 | 1.10 (1.01; 1.20) | 0.085 |  |
| ≥ median | 3,265 | 661 | 1.06 (0.98; 1.15) | 0.200 |  |
| **25 (OH)D levels ^f^** |  |  |  |  | 0.063 |
| Deficient | 893 | 180 | 1.08 (0.94; 1.25) | 0.349 |  |
| Insufficient | 2,452 | 470 | 1.04 (0.95; 1.14) | 0.454 |  |
| Sufficient | 3,184 | 585 | 1.13 (1.04; 1.23) | 0.077 |  |

Abbreviations: 25-hydoxyvitamin D, 25(OH)D; BMI, body mass index; CI, confidence interval; CRP, C-reactive protein; HDL-C, high density lipoprotein cholesterol; HbA_1c_: hemoglobin A_1c_; HR, hazard ratio; Q, quintiles; SELENOP, selenoprotein P; TG, triglyceride.

^a^ HR for per 1 SD increment of serum SELENOP concentrations. Models were adjusted for age and sex, education, family history of diabetes, systolic blood pressure, BMI, smoking status, smoking pack-years, physical activity, regular multivitamin supplement use, dietary intake of fruit, dietary intake of vegetables, lipid-lowering medication, antihypertensive medication, HbA_1c_, TG to HDL-C ratio, albumin, CRP, 8-isoprostane, 25(OH)D concentrations and season of blood draw.

^b^ Interaction analyses were conducted by adding an interaction term (continuous serum SELENOP concentration × sex/ age groups/ continuous BMI/ smoking status groups/ physical activity groups/ prediabetes groups/ continuous CRP/ continuous 8-isoprostane/ continuous 25 (OH)D levels) to the multivariable Cox regression models.

^c^ “inactive” or “low”, < 2 hours each of vigorous and light physical activity per week; or “medium or high”, ≥ 2 hours per week of both.

^d^ No, HbA_1c_ <5.7%; or Yes, HbA_1c_ 5.7-6.4%.

^e^ No, CRP <3 mg/L; Yes, CRP ≥3 mg/L.

^f^ Deficient, 25(OH)D <30 nmol/L; insufficient, 25(OH)D 30 to <50 nmol/L; or sufficient, 25(OH)D ≥50 nmol/L.
